# Tracking Peripheral Memory *T* Cell Subsets in Advanced Nonsmall Cell Lung Cancer Treated with Hypofractionated Radiotherapy and PD-1 Blockade

**DOI:** 10.1155/2023/3221510

**Published:** 2023-01-31

**Authors:** Pengyuan Kang, Hong Yu, Yunfei Li, Xue Wen, Hua Ye, Yuhao Luo, Yaqi Yang, Qing Yuan, Sheng Lin

**Affiliations:** ^1^Department of Oncology, The Affiliated Hospital of Southwest Medical University, Luzhou, Sichuan Province 646000, China; ^2^Public Center of Experimental Technology, The School of Basic Medical Sciences, Southwest Medical University, Luzhou, Sichuan Province 646000, China; ^3^Department of Laboratory Medicine, The Affiliated Hospital of Southwest Medical University, Luzhou, Sichuan Province 646000, China; ^4^Nuclear Medicine and Molecular Imaging Key Laboratory of Sichuan Province; Institute of Neclear Medicine, Southwest Medical Universty, Luzhou 646000, China; ^5^Academician (Expert) Workstation of Sichuan Province, Sichuan, Luzhou, China

## Abstract

Hypofractionated radiotherapy (HFRT) or chemotherapy combined with programmed death-1 (PD-1) blockade has achieved good clinical control in advanced nonsmall cell lung cancer (NSCLC). However, the relative influence of HFRT + PD-1 blockade and chemo-immunotherapy on peripheral memory T cell subsets in NSCLC responders has not been evaluated in clinical practice. Thirty-nine patients with advanced NSCLC were enrolled. The frequencies of naive (Tn; CD45RA^+^CCR7^+^), central memory (Tcm; CD45RA^–^CCR7^+^), effector memory (Tem; CD45RA^–^CCR7^–^), and effector memory RA (TemRA; CD45RA^+^CCR7^–^) T cell subsets and PD-1 expression were analyzed in CD4^+^ and CD8^+^ T cells using flow cytometry from peripheral blood samples. The correlations of memory T cell subsets and PD-1 expression with overall survival in HFRT + PD-1 blockade group were examined using the Kaplan–Meier method. Patients with partial response to HFRT + PD-1 blockade showed reduction in Tn and expansion in TemRA cell subpopulations among CD8^+^ T cells and reduced PD-1^+^CD4^+^ and PD-1^+^CD8^+^ T cells, all of which were significantly correlated with overall survival. The responders to chemo-immunotherapy showed expansion of the TemRA and decrease of Tcm in CD8^+^ T cell subpopulation. Our findings show that HFRT+PD-1 blockade and chemo-immunotherapy combination therapies induce differential memory T cell subset differentiation, offering predictive markers for treatment response. Clinical Trial Information: https://clinicaltrials.gov/ct2/show/ChiCTR-1900027768.

## 1. Introduction

The development of immunotherapy-based treatment combinations represents a significant milestone in NSCLC treatment [1]. The success of immunotherapy has now driven a paradigm shift in the treatment of advanced NSCLC, combined therapy promotes the formation of immune memory. In particular, chemotherapy combined with PD-1 blockade has been shown to promote immune cell infiltration in the tumor microenvironment and mediate the peripheral immune memory cell phenotype [2–4]. Immunotherapy based on programmed death-1 (PD-1) has improved clinical outcomes in patients with nonsmall cell lung cancer (NSCLC), however, the efficiency of monotherapy remains low, resulting in increased number of trials of combined therapies, with encouraging results [5]. Therefore, more studies are needed to explore the discrepancy in memory T cell subsets among different combination therapies.

Immune cells play an important role in suppressing or promoting tumor development, metastasis, and progression [6]. Immune memory is essential for long-term immunity and is a key factor for the long-term benefit of tumor immunotherapy [7]. Circulating T cells with tumor antigen specificity are widely found in patients with cancer [8]. Once the organism encounters a viral infection or tumorigenesis, naive T cells are activated by antigens, and some effector T cells become long-lived memory T cells to initiate immune effects [9]. According to anatomical location and phenotypic characteristics, naive T cells (Tn; defined as CD45RA^+^CCR7^+^) differentiate into central (Tcm; CD45RA^–^CCR7^+^) and effector (Tem; CD45RA^–^CCR7^–^) memory T cells upon antigen activation. Another terminal subpopulation expressing CD45RA (TemRA) was also recently identified [10, 11].

Irradiation can alter tumor-host interactions and restore tumor immunogenicity [12], leading to dynamic changes in peripheral blood lymphocyte ratios [13]. Stereotactic body radiation therapy (SBRT) can stimulate innate and adaptive immunity and thus improve the efficacy of tumor immunotherapy [14, 15]. Evaluation of endogenous antigen-specific immune responses triggered by SBRT and PD-1 blockade showed that radiotherapy increases the differentiation of antigen-experienced T cells to Tem cells [16]. The prescribed doses of SBRT are designed to wrap around the planning target volume (PTV) as much as possible while prioritizing meeting organ-threatening dose limits. In contrast, hypofractionated brachytherapy (HFBT) uses a ^192^Ir source and prescribes doses that wrap around the gross tumor target volume (GVT) as much as possible without outgrowing the clinical target dose volume and PTV under the premise of prioritizing organ-threatening doses. However, the relative influence of HFRT + PD-1 blockade and chemo-immunotherapy on peripheral memory T cell subsets in NSCLC responders has not been evaluated in clinical practice.

In our previous study, the overall objective response and complete remission rate were 39.13% and 13.04%, respectively [17]. CR patients maintained continuous remission for a 2-yearfollow-up period. The aim of the current study was to investigate the differentiation of memory T cells in NSCLC responders treated with HFRT + PD-1 and to explore the differences in memory T cell subsets with those induced by chemo-immunotherapy. We hope this study could bring better clinical treatment implications for NSCLC patients.

## 2. Methods and Materials

### 2.1. Study Design and Treatment

This prospective trial was approved by our ethics committee (No. KY2019276). Thirty-nine patients with advanced NSCLC were enrolled in the trial. The study was conducted in accordance with the principles of the 1964 Declaration of Helsinki and its subsequent amendments. We followed the methods of Kang et al., and the detailed medication doses and radiotherapy protocols are available in this report [18].

### 2.2. Specimen Collection

A prospective collection of peripheral blood samples from 39 patients with NSCLC who met the inclusion criteria and were enrolled in the study between May 2019 and July 2021, and then again at 3 months after treatment (Table 1). Samples collected before any HFRT + PD-1 blockade were considered baseline, and samples were collected at least 1 week after completion of radiotherapy after HFRT + PD-1 blockade, only samples from patients before their first radioimmunotherapy or chemo-immunotherapy were included in the comparison between the healthy donor and patient groups. Single-cell suspensions were prepared using Ficoll-Paque gradient centrifugation within 24 h after anticoagulation of fresh blood with EDTA-K2. A programmed gradient was used to freeze then thawed at various time points to assess the frequency of T cell subpopulations. Healthy control samples were obtained from 20 donors matched to the age and sex distributions of the patients.

### 2.3. Assessment of Tumor Volume and Clinical Response

Clinical response was assessed using computed tomography (CT) scans according to the Response Evaluation Criteria for Solid Tumors (RECIST V1.1) and classified as partial response (PR), stable disease (SD), or progressive disease (PD). Further details are provided in our previous report [17]. Interpretation of response from positron emission tomography (PET) scans was based on the European Organization for Research and Treatment of Cancer 1999 standard [19].

### 2.4. Flow Cytometry

Cells were washed with 10% fetal bovine serum +90% RPMI1640 medium; after being washed with phosphate-buffered saline, the cells were treated with human Trustain FCX specific antibodies to block nonspecific binding. For extracellular staining, 1 × 10^6^ isolated peripheral blood mononuclear cells (PBMCs) were stained with CD3-APC/Cyanine7 (300318; Biolegend USA), CD4-FITC (300506; Biolegend USA), CD8-PerCP/Cyanine5.5 (344710; Biolegend USA), CD279-APC Clone MIH4 (RUO) (558694, BD, USA), CD45RA-Brilliant Violet510 (304142; Biolegend USA), and CD197(CCR7)-Brilliant Violet421 (353208; Biolegend USA). After being washed with phosphate-buffered saline, the cells were collected by flow cytometry (BD, FACS CantoII, USA) and analyzed using BD FACS Diva software for circle gate analysis.

### 2.5. Statistical Analysis

Overall survival (OS) was defined as the time from the first fraction of HFRT to death from any cause or the last follow-up visit, estimated using the Kaplan–Meier method. All quantitative variables are expressed as mean ± standard or median deviation. For comparison of median differences between the different response groups, the Mann–Whitney U test was used. All statistical analyses were performed using SPSS Version 17.0 software (SPSS, Inc., Chicago IL, USA) and GraphPad Prism 8 (GraphPad Software Inc. USA). Cut-off values for high and low OS were determined using receiver operating characteristic (ROC) curve. *p* values were calculated using paired *t*-test and independent samples *t*-test. Differences were considered significant with a *p* value below 0.05.

## 3. Results

### 3.1. Differences in Peripheral Circulating T Cells in NSCLC Patients and Healthy Individuals

**Figure 1:**
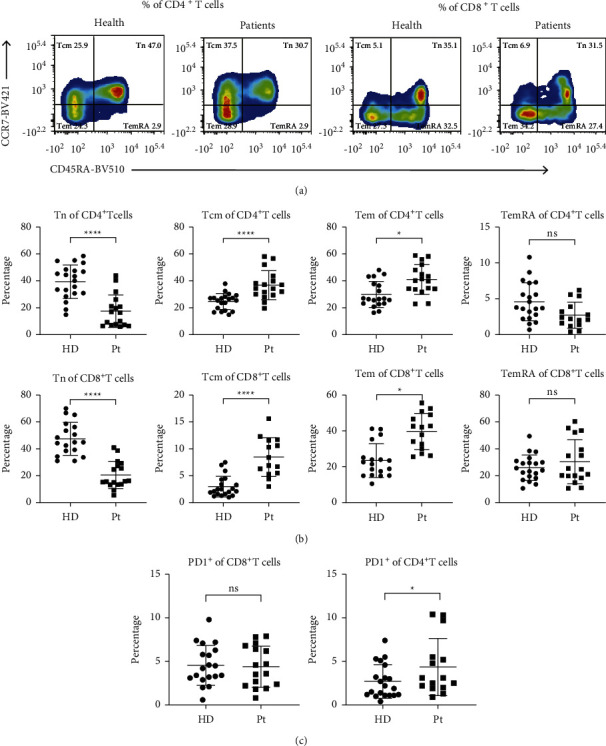
Peripheral immune memory T cell subsets in untreated NSCLC patients (*n* = 17) show stronger immune memory effects than those in healthy donors (*n* = 20). (a) Gating scheme for memory T cell subpopulations in PBMCs. (b) Differences in naïve (Tn), central memory (Tcm), effector memory (Tem), and effector memory RA (TemRA) T cells among CD4^+^ and CD8^+^ T cells between NSCLC patients and healthy controls. (c) CD4^+^PD-1^+^ and CD8^+^PD-1^+^ T cell subpopulations among PBMCs from NSCLC patients and healthy controls. HD, healthy donors; Pt, patients. Data are shown as the mean ± SD, ^*∗*^*p*  <  0.05, ^*∗∗*^*p*  <  0.01, ^*∗∗∗*^*p*  <  0.001, ^*∗∗∗∗*^*p*  <  0.001, and *ns,* not significant.

We analyzed T cell subsets in PBMCs from 17 untreated NSCLC patients and 20 healthy donors.  Figure 1(a) shows representative flow cytograms indicating the percentages of memory T cell subpopulations in one healthy donor and one patient. The TemRA population was predominantly present in the CD8^+^ T cell subpopulation, and the percentage of Tn cells among both CD4^+^ and CD8^+^ T cells was lower in NSCLC patients than in healthy controls (*p*  <  0.0001), whereas those of Tcm (*p*  <  0.0001) and Tem (*p*=0.013, *p*=0.017) cell subpopulations were elevated in patients (Figure 1(b)), which is consistent with the findings of a previous study [20]. The expression of PD-1 on CD4^+^ T cells was remarkably higher in NSCLC patients than in healthy controls (*p*=0.037), whereas its expression on CD8^+^ T cells did not differ significantly between the two groups (Figure 1(c)).

### 3.2. Expansion of TemRA Cell Subpopulation and Decreased PD-1 Expression Associated with PR after HFRT + PD-1 Blockade

**Figure 2:**
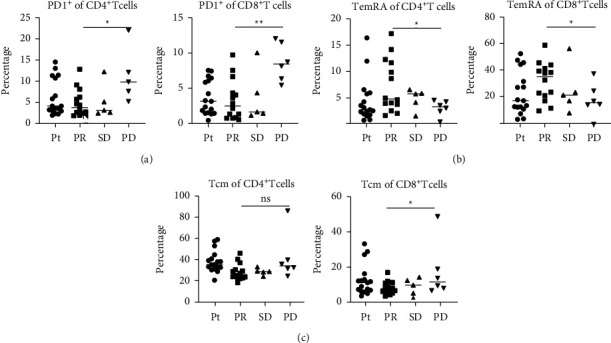
Differences in memory T cell subpopulations in different responder patient groups after treatment with HFRT + PD-1 blockade. (a) Differential expression of PD-1 on CD4^+^ and CD8^+^ T cells in patient, NSCLC patient (Pt), partial response (PR), stable disease (SD), and progressive disease (PD) groups. Differences in TemRA (b) and Tem (c) cell subpopulation frequencies and among CD4^+^ and CD8^+^ T cells in the PR, SD, and PD groups. Data are shown as the median, ^*∗*^*p*  <  0.05, ^*∗∗*^*p*  <  0.01, ^*∗∗∗*^*p*  <  0.001, ^*∗∗∗∗*^*p*  <  0.001, and ns, not significant.

According to RECIST v1.1, 14 patients exhibited PR, 5 exhibited SD, and 6 exhibited PD at follow-up. The PR group showed a trend of decreased frequency of CD4^+^ PD-1^+^ and CD8^+^ PD-1^+^ T cell subsets (Figure 2(a)) (*p*=0.019, *p*=0.003). Moreover, disease progression was associated with less TemRA (Figure 2(b)) (*p*=0.013, *p*=0.038) and expansion of the Tcm cell subpopulation (Figure 2(c)) (*p*=0.037), There was no significant difference in the Tn and Tem cell subpopulations between the groups. And we compared the different groups of pretreatment finding that there were no significant differences between them.

### 3.3. Expansion of TemRA Cell Subpopulation after HFRT + PD-1 Blockade in PR Patients

**Figure 3:**
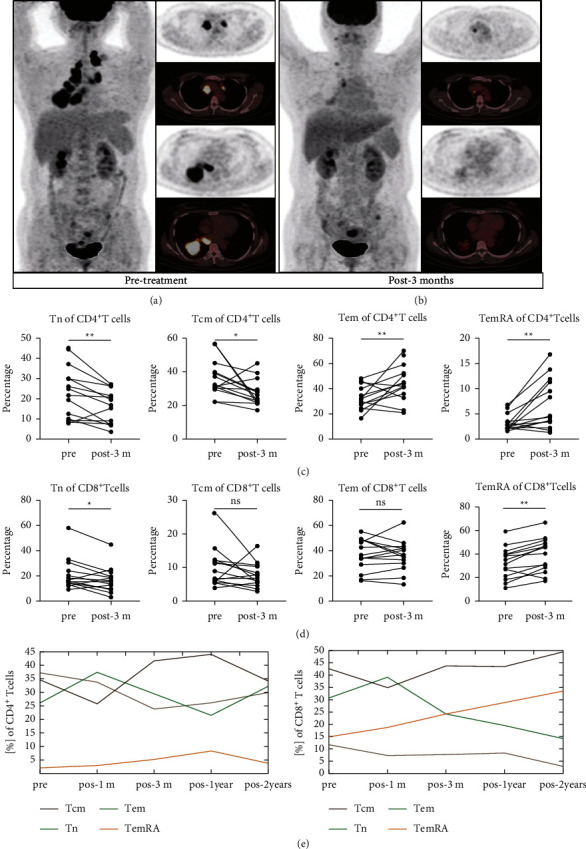
Imaging data and changes in immune memory T cell subsets in patients who showed partial response (PR) before and after treatment with HFBT + PD-1 blockade. (a, b) PET-CT fusion images and PET tomography data of a PR patient treated with HFBT + PD-1 blockade showing excellent tumor control. Comparison of peripheral CD4^+^ (c) and CD8^+^ (d) memory T cell subsets in PR patients pre- and post-treatment with HFRT + PD-1 blockade. (e) Trends of peripheral memory T cell subsets over 2 years in a patient with long-term PR. PET-CT: positron emission tomography-computed tomography. ^*∗*^*p*  <  0.05, ^*∗∗*^*p*  <  0.01, ^*∗∗∗*^*p*  <  0.001, ^*∗∗∗∗*^*p*  <  0.001, and *ns,* not significant.

CT images and PET-CT imaging data of a PR patient before and after treatment with SBRT combined with PD-1 blockade demonstrated excellent tumor control after treatment with HFRT (Figures 3(a) and 3(b)). For the PR group, compared with the pretreatment levels, the post-treatment Tn and Tcm subpopulations in CD4^+^ T cells were significantly diminished (*p*=0.009, *p*=0.041), whereas the Tem and TemRA cell subsets were significantly expanded (Figure 3(c)) (*p*=0.004, *p*=0.003). As the primary killing cells, the CD8^+^ T cell subpopulation showed a significant decrease in the naïve T cell subpopulation and expansion in the TemRA cell subpopulation (Figure 3(d)) in the PR group (*p*=0.026, *p*=0.005), which is similar to the findings of Kunert et al. [21], CD8^+^ T cell populations in PR patients show enhanced frequencies of TemRA cell compared to PD patients at baseline and during treatment. Since HFRT and PD-1 blockade showed good efficacy and elicited long-term survival in several patients in this trial, all of whom survived for at least 2 years, we further analyzed the T cell subsets of PBMCs in these long-term remission patients to obtain insight into the potential long-term presence of memory T cells, there was a trend of reduction of Tn subpopulation and a gradually rising trend of TemRA in CD8^+^ T cells (Figure 3(e)).

### 3.4. Differences in Memory T Cell Subsets before and after Chemo-Immunotherapy

**Figure 4:**
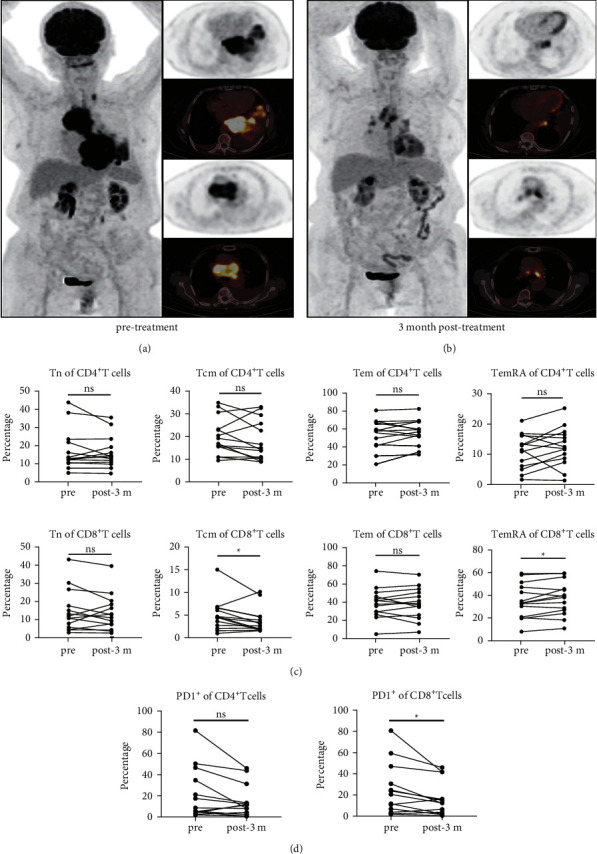
Imaging data and changes in immune memory T cell subsets in partial response (PR) patients before and after chemo-immunotherapy. (a, b) PET-CT fusion images and PET tomography data of a patient with PR after chemo-immunotherapy treatment showing good tumor control. (c) Comparison of peripheral CD4^+^ and CD8^+^ memory T cell subsets in PR patients pre- and post-treatment with chemo-immunotherapy. (d) Differential expression of PD-1 on CD4^+^ and CD8^+^ T cells in PR with chemo-immunotherapy. PET-CT: positron emission tomography-computed tomography. ^*∗*^*p*  <  0.05, ^*∗∗*^*p*  <  0.01, ^*∗∗∗*^*p*  <  0.001, ^*∗∗∗∗*^*p*  <  0.001, and *ns,* not significant.

CT and PET-CT imaging data of a PR patient before and after chemotherapy combined with immunotherapy are shown in Figures 4(a) and 4(b), demonstrating that the tumor lesions were effectively cleared. Flow cytometry of T cell subsets from PBMCs in the PR group that received chemo-immunotherapy (*n* = 14) showed significant reduction in Tcm subpopulation (*p*=0.044) and the TemRA subpopulation expanded after treatment in CD8^+^ T cells (Figure 4(c)) (*p*=0.048). PD-1 expression in CD8^+^ T cells decreased after chemo-immunotherapy (Figure 4(d)) (*p*=0.011), while there was no significant discrepancy in the CD4^+^ T cells (*p*=0.07).

### 3.5. Association of Memory T Cell Subsets and PD-1 Expression with Survival in HFRT + PD-1 Blockade Group

**Figure 5:**
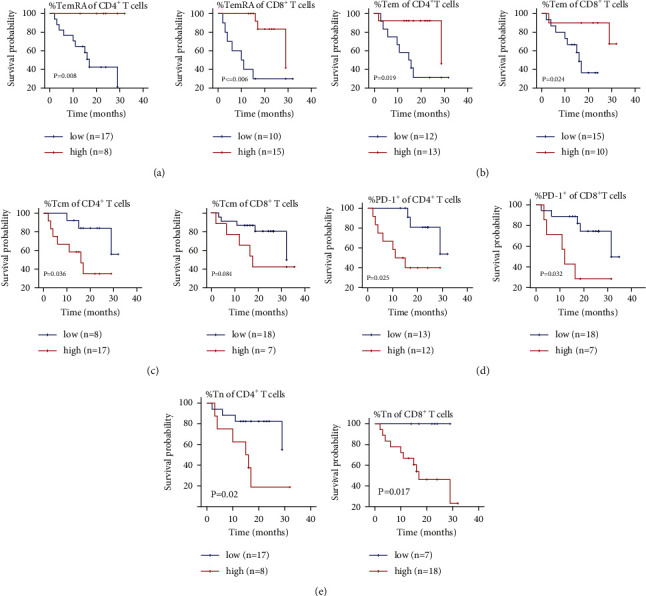
Elevated effector memory T cell count and low expression of PD-1 associated with high survival rates in NSCLC patients treated with HFRT + PD-1 blockade. Expanded effector memory RA (TemRA) (a) and effector memory (Tem) (b) T cell subpopulations, reduced naïve (Tn) (c) and central memory (Tcm) (d) T cell subpopulations, and low expression of PD-1 (e) in NSCLC patients treated with HFRT are associated with high survival rates.

**Figure 6:**
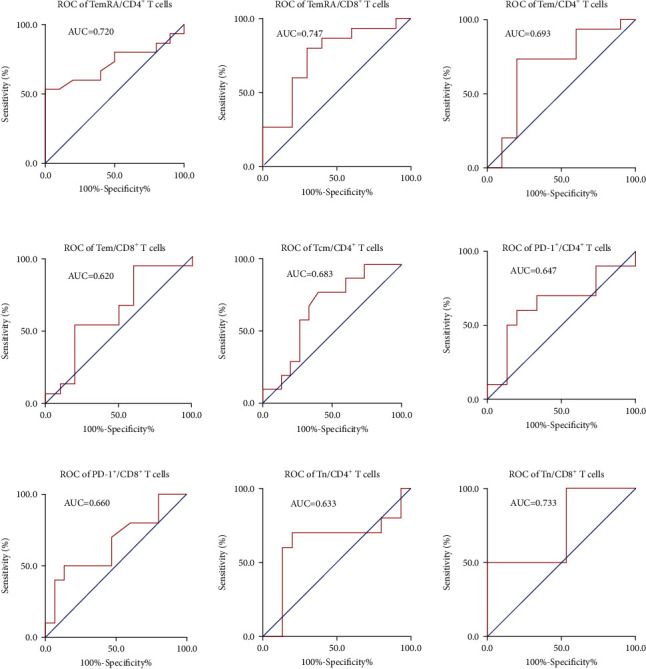
Receiver operating characteristic curves to assess predictive ability of immune T cell subpopulations to discriminate between high and low survival probability, as shown in Figure 5.

An expanded TemRA or Tem cell subpopulation among CD4^+^ and CD8^+^ T cells was positively associated with OS (Figures 5(a) and 5(b)). In contrast, a small Tcm (CD45RA^–^CCR7^+^) cell subpopulation among CD4^+^ T was positively correlated with OS (Figure 5(c)). Similarly, reduced PD-1 expression in both CD4^+^ T and CD8^+^ T cells was associated with longer OS (Figure 5(d)). In addition, based on the pretreatment data, a reduced Tn (CD45RA^+^CCR7^+^) cell subpopulation among CD4^+^ T and CD8^+^ T cells was found to prospectively predict better survival (Figure 5(e)). These findings further support that activation of memory T cell subsets has a favorable prognosis. The ROC curve clearly distinguished memory T cell subpopulations according to OS (Figure 6). The most sensitive and specific indicator of OS was the TemRA cell subpopulation among CD8^+^ T cells, with an area under the curve (AUC) value of 0.747, followed by the Tn cell subpopulation among CD8^+^ T cells (AUC = 0.733). TemRA cell subpopulation among CD4^+^ T cells (AUC = 0.720), Tem cell subpopulations among CD4^+^ T and CD8+ T cells (AUC = 0.693 and 0.620, respectively), and Tcm cell subpopulation among CD4^+^ T cells (AUC = 0.683). The AUC values for PD-1 expression of CD4^+^ and CD8^+^ T cells were 0.647 and 0.683, respectively. The AUC of Tn cells among CD4^+^ T cells before treatment was 0.633 and that of CD8^+^ T cells after treatment was 0.733.

## 4. Discussion

In this study, we evaluated four subpopulations (Tn, Tcm, Tem, and TemRA) of memory T cells among PBMCs, which were found to be distinctly activated and differentiated in NSCLC patients compared with those in healthy controls. In particular, the frequency of TemRA cells was markedly increased, and PD-1 expression was decreased in the PR patient group after HFRT + PD-1 blockade and chemo-immunotherapy, suggesting distinct mechanisms of response to different combination treatments, these results are consistent with evidence that increased PD-1 expression is associated with poor prognosis [20]. Specifically, these results suggested that the naïve T cell subpopulation in NSCLC patients differentiates toward the effector memory T cell subpopulation after antigen stimulation, and high expression of PD-1 on CD4^+^ T cells may be responsible for the suppression of antitumor effects [22].

Radiotherapy combined with immunotherapy can increase the percentage of effector memory T cells that can stimulate endogenous antigen-specific immune responses in advanced NSCLC, thereby enhancing the immunotherapeutic response [15, 23, 24]. We also found a significantly higher frequency of Tcm cells in both CD4^+^ and CD8^+^ T cell subpopulations in our NSCLC cohort compared with that in the healthy controls. Together, these results indicate that immune effects were activated in NSCLC, thereby validating the association between expanded Tcm cell subpopulation and enhanced tumor inflammatory features [25, 26]. Moreover, as an indicator of disease progression [27], PD-1 expression of the PD group was significantly higher than the PR group, which might reflect the better response to PD-1 blockade in the latter group.

Memory cells are abundant in responders to combination immunotherapy [28], and CD8^+^ T effector cell memory subpopulations show the major T cell phenotypic expansion in patients responding to treatment [29]. A key factor in the generation of memory CD8^+^ T cells is the help provided by CD4^+^ T cells [30]. We identified the presence of four memory cell subpopulations among both CD4^+^ and CD8^+^ T cells, although these subpopulations were mainly present in CD8^+^ T cells. During the differentiation of memory T cells, Tn cells are activated as an abundant initial subpopulation and then differentiate into central memory cells as well as effector memory cells [31–34], along with the terminally differentiated subpopulation expressing CD45RA (TemRA) [10]. TemRA cells in CD4^+^ T cells have been reported to be associated with protective immunity against pathogens such as dengue virus [11], and TemRA cells among CD8^+^ T cells can generate rapid responses to stimuli, playing a specific role in immune surveillance with high proliferative capacity and differentiation plasticity [35, 36], which has challenged the conventional view that the TemRA cell subpopulation is primarily associated with immune system aging [37, 38]. We also found a decrease in the initial memory cell subpopulation and a significant expansion of the TemRA cell subpopulation in the PR group of patients with HFRT + PD-1 blockade maintenance for 2 years. These results suggested that a better treatment response is associated with immune memory function activated by stimulation, and the memory T cell subpopulation gradually differentiates toward effector memory cells that perform the killing function [30]. As the storage pool of memory cells [25], we found a reduction in the Tcm cell subpopulation among CD4^+^ T cells but expansion of the Tem cell subpopulation, suggesting that memory T cells show differentiation from naïve memory T cells to the memory effector subpopulation in patients with better response to treatment. Moreover, the central circulating pool of cells was continuously replenished in the CD8^+^ T cell subpopulation. Furthermore, among the PR, SD, and PD groups, the TemRA subpopulation was diminished in the PD patient group, whereas that of Tcm cells was expanded mainly among CD8^+^ T cells, which indicates that the patients in the PD group did not have fully functional memory clearance because the TemRA cell subpopulation was not sufficiently formed.

Chemotherapy combined with immunotherapy has shown good efficacy in the treatment of advanced NSCLC [39, 40], enhancing memory cell phenotypes [2], and peripheral memory T cells are associated with clinical prognosis, as found after SBRT [41]. However, differences in the frequencies of memory T cell subpopulations between the two treatments have been rarely reported, thus, we investigated changes of memory T cell subpopulations in PR patients after chemo-immunotherapy, revealing only significant reduction in the Tcm subpopulation among CD8^+^ T cells. This suggests that peripheral memory T cells show different patterns of activation according to different types of combination therapies.

Our data also demonstrated the association of OS with memory T cell subsets in the entire cohort of NSCLC patients treated with HFRT + PD-1, with increased frequencies of the TemRA and Tem cell subsets significantly associated with longer survival, whereas the Tn and Tcm cell subpopulations showed the opposite trend. PD-1 expression was negatively correlated with OS, and the ability of these subgroups to predict OS was reflected in the ROC curve.

Nevertheless, there are some limitations of our study that should be mentioned. In particular, the sample size was small, and peripheral blood sampling was performed before and after curative effect assessment rather than at an absolute fixed time point, and patients in the chemo-immunotherapy group have not a long enough OS to be evaluated, these might lead to bias in the conclusions.

In conclusion, this study demonstrated that HFRT and chemotherapy combined with PD-1 blockade can trigger differentiation of memory T cells in responders. The TemRA cell subpopulation was highly expanded in patients showing treatment response and long-term remission, and correlated with OS, indicating its potentially predictive value in the response to HFRT + PD-1 treatment. The different patterns of immune memory cell differentiation between HFRT + PD-1 and chemo-immunotherapy suggest that sequential radio-chemo-immunotherapy could promote comprehensive stimulation of immune memory, and thus might bring greater clinical benefit to patients with NSCLC.

## Figures and Tables

**Table 1:** Baseline characteristics of patients.

Characteristics	No.
*Age (year)*	

Median (range)	58 (38–72)

*Gender*	

Male	27
Female	12

*ECOG status*	

0	10
1	18
2	11

*Smoking status*	

Former and current	19
Never	20

*Histologic type*	

Squamous	14
Adenocarcinoma	23
Other NSCLC	2

*Staging*	

IIIA	7
IIIB	6
IIIC	3
IVA	12
IVB	11

*Follow duration (mo)*	

Median (range)	14 (2–32)

*Combination with PD-1*	

SBRT or SABR	25
No SBRT or SABR	14

ECOG, Eastern Cooperative Oncology Group; SBRT, stereotactic body radiotherapy; SABR, stereotactic ablative radiotherapy; mo, month; fx, fraction.

## Data Availability

The data presented in this study are available on request from the corresponding author.
